# The relationship between fleas and small mammals in households of the Western Yunnan Province, China

**DOI:** 10.1038/s41598-020-73690-0

**Published:** 2020-10-07

**Authors:** Jia-Xiang Yin, Xiao-Ou Cheng, Yun-Yan Luo, Qiu-Fang Zhao, Zhao-Fei Wei, Dan-Dan Xu, Meng-Di Wang, Yun Zhou, Xiu-Fang Wang, Zheng-Xiang Liu

**Affiliations:** 1 School of Public Health, Dali University, Dali, 671000 Yunnan Province People’s Republic of China; 2 Yunnan Institute of Endemic Disease Control and Prevention, Dali, 671000 Yunnan Province People’s Republic of China

**Keywords:** Zoology, Medical research

## Abstract

The Yunnan province has one of the most serious outbreaks of the plague epidemic in China. Small mammals and fleas are risk factors for the occurrence of plague in commensal plague foci. Understanding the relationship between fleas and small mammals will help control fleas and prevent the onset of the plague. Four hundred and twenty-one small mammals, belonging to 9 species, were captured. Of these, 170 small mammals (40.4%) were found infested with fleas. A total of 992 parasitic fleas (including 5 species) were collected. The number of *Leptopsylla segnis* and *Xenopsylla cheopis* accounted for 91.03% (903/992). The final multiple hurdle negative binomial regression model showed that when compared with *Rattus tanezumi*, the probability of flea infestation with *Mus musculus* as well as other host species decreased by 58% and 99%, respectively, while the number of flea infestations of the other host species increased by 4.71 folds. The probability of flea prevalence in adult hosts increased by 74%, while the number of fleas decreased by 76%. The number of flea infestations in small male mammals increased by 62%. The number of fleas in small mammals weighing more than 59 g has been multiplied by about 4. *R. tanezumi* is the predominant species in households in the west Yunnan province, while *L.segnis* and *X. cheopis* were dominant parasitic fleas. There is a strong relationship between the abundance of fleas and the characteristics of small mammals (e.g. Species, age, sex, and body weight).

## Introduction

Plague is a zoonotic bacterial infectious disease transmitted by fleas with reservoirs in rodent populations, in which fleas play a potentially fundamental role as bridge vectors to transmit the bacteria (*Yersinia pestis*) to animals and humans^1^. The potential role of fleas as carriers and host-supported local reservoirs can help explain the persistent occurrence of plague^2^.

The plague has played a huge role in human history and is still prevalent in most parts of China. In China, animal plague is reported almost every year, and occasional human plagues did occur^3,4^. Transmission to humans sometimes occurs through contact with fleas that have fed on an infected small mammal or by skinning infected small mammals (e.g. *Marmot*)^5–7^. The province of Yunnan is one of the most serious plague epidemic foci in China^8,9^, and especially the most active in commensal rodent plague foci^2^. The province of Yunnan is characterized by rich varieties of natural conditions, which high in the north and low in the south of the terrain, spanning 8 latitude zones and possessing a rich geographical landscape. Also it possesses an obvious vertical climate and incorporates in cold, temperate and tropical, consisting of embedded microhabitats that create a series of ecological niches. The above all increased possibility survival and reproduction of small mammals and fleas and risk spread and preservation of *Y. pestis* in them^10^.

Adults fleas are obligatory hematophagous insects and rodents are the most common host of fleas, although they can appear in other types of mammals and birds^11,12^. At different stages of the life cycle, fleas are located in the hosts or in the host's nest. In most cases, the imago flea is usually parasitic on the host and until the end of its life cycle, differentiating with the larvae. When leaving a pupa, a new flea of ​​imago finds a host because the reproduction of most fleas is not possible without the feeding of blood^13^. Therefore, it is likely to handle flea infestations on live hosts. The parasites get their food and other biological supplies from their hosts. Consequently, they can affect the host directly by reducing host resources, and indirectly by provoking behavioral responses^14,15^ or immunological responses^1,16^. Fleas with high density and richness not only favor the bacteria plague, but also spread widely among the hosts, and also suppress the immune response of the hosts, thus improving the susceptibility of the hosts^17,18^, which is vital for the maintenance of the plague^19,20^.

The abundance of hosts is an important factor that affects the distribution and abundance of parasites. In terms of the reservoir, some individuals, populations or host species are characterized by a higher level of parasite infestation than others^21^. It is well known that there is a great variation in the abundance of a parasite among the different host species. The host with the highest abundance of parasites is commonly defined as the main host, while those with lower abundance of parasites are defined as auxiliary hosts^21,22^.

In this study, we have attempted to describe the distribution of small mammals and fleas and to examine the effect of small mammals on parasites abundance in the western province of Yunnan. The information on the factors that affect the flea abundance in hosts is essential to provide evidence-based recommendations on flea control and to implement plague control and prevention programs in plague natural foci.

## Results

### Distribution and description of small mammals

A total of 12,000 traps were placed in 800 households in 40 villages from 10 counties in the western Yunnan province and 421 small mammals were captured. The overall density of small mammals was 3.51%. Small mammals were divided into 9 species, 6 genera, 2 families and 2 orders (Table 1). *Rattus tanezumi* is the dominant species and it represents 66.03% (278/421) of the entire sample. Among the 10 counties, the highest number and the smallest number of small mammals were captured in Mangshi (100) and Deqin (16) counties, respectively. The number of small mammals richness was 9. The highest number and lowest number of richness were in Yongren (5), Nanhua (1) and Deqin (1) counties, respectively. (Table 1).

**Table 1:** Distribution of small mammals in households from 10 counties in the western Yunnan, China.

County	Small mammals			Species and proportion (%) of small mammals								
	Abundance	Density	Richness	*R. tanezumi*	*M. musculus*	*Rattus norvegicus*	*Rattus nitidus*	*Suncus murinus*	*Apodemus chevrieri*	*Crocidura attenuata*	*Anoro sorex squamipes*	*Mus caroli*
Yongren	86	7.17	5	4 (4.65)	73 (84.88)	2 (2.33)	6 (6.98)	0	0	0	0	1 (1.16)
Nanhua	36	3.00	1	36 (100.00)	0	0	0	0	0	0	0	0
Xiangyun	41	3.42	3	18 (43.90)	0	22 (53.66)	0	1 (2.44)	0	0	0	0
Yunxian	39	3.25	2	37 (94.87)	2 (5.13)	0	0	0	0	0	0	0
Gengma	26	2.17	2	25 (96.15)	1 (3.85)	0	0	0	0	0	0	0
Mangshi	100	8.33	2	94 (94.00)	0	0	0	6 (6.00)	0	0	0	0
Longyang	28	2.33	3	24 (85.71)	0	0	0	0	0	2 (7.14)	2 (7.14)	0
Lanping	22	1.83	3	17 (77.27)	0	4 (18.18)	1 (4.55)	0	0	0	0	0
Yulong	27	2.25	3	23 (85.18)	0	2 (7.41))	0	0	2 (7.41)	0	0	0
Deqin	16	1.33	1	0	0	0	16 (100.00)	0	0	0	0	0
Total	421	3.51	9	278 (66.03)	76 (18.05)	30 (7.13)	23 (5.46)	7 (1.66)	2 (0.48)	2 (0.48)	2 (0.48)	1 (0.24)

^a^Small mammals density = (abundance/ live traps.nights) × 100%, the number of live traps.nights each county is 1200.

In this study, the weight, body length and tail length of 407 small mammals were calculated (for other 14 small mammals there was no calculation due to incomplete data). The mean body weight was 62.93 g, standard deviation 42.67 g, median 59.31 g (range 7.39–186.89 g); the average body length was 22.79 cm, the standard deviation of 8.57 cm and the median 21.00 cm (range 7.50–39.30 cm); the average length of the tail was 12.30 cm, the standard deviation of 3.76 cm and the median 13.30 cm (extremes: 1.00 and 20.00 cm).

### Distribution of parasitic fleas

Of 421 small mammals, 170 small mammals were infested with fleas and their prevalence was 40.38%. A total of 992 fleas, divided into 5 species, 5 genera, and 3 families, were collected and the flea index was 2.36 (992/421). The highest flea prevalence and the highest flea index occurred in Yunxian county (71.79%) and Yulong county (7.33), respectively. Out of 992 fleas, the number of *Xenopsylla cheopis* and *Leptopsylla segnis* was 903, which accounted for 91.03%. The proportion of *X. cheopis* exceeds 90% in Yunxian and Gengma counties (Table 2).

**Table 2:** Distribution of parasitic fleas in households in 10 counties from the western Yunnan, China.

County	No. of small mammals	No. of small mammals with flea infection	Flea prevalence (%)^a^	No. of fleas	Flea index^b^	Richness	Parasitic flea species and proportions				
							*X. cheopis*	*L. segnis*	*Ctenocephalides felis felis*	*Monopychllusanisus*	*Pulexirritans*
Yongren	86	6	6.98	83	0.97	3	71 (85.54)	11 (13.26)	0	1 (1.20)	0
Nanhua	36	12	33.33	86	2.39	4	1 (1.16)	61 (70.93)	8 (9.3)	0	16 (18.6)
Xiangyun	41	21	51.22	146	3.56	2	3 (2.05)	143 (97.95)	0	0	0
Yunxian	39	28	71.79	97	2.49	2	92 (94.85)	5 (5.15)	0	0	0
Gengma	26	12	46.15	42	1.62	2	38 (90.48)	0	0	4 (9.52)	0
Mangshi	100	56	56.00	250	2.50	3	208 (83.20)	34 (13.60)	0	8 (3.20)	0
Longyang	28	11	39.29	41	1.46	4	16 (39.02)	23 (56.09)	1 (2.44)	1 (2.44)	0
Lanping	22	9	40.91	49	2.23	2	0	45 (91.84)	0	4 (8.16)	0
Yulong	27	15	55.56	198	7.33	3	0	152 (76.77)	35 (17.68)	11 (5.56)	0
Deqin	16	0	0	0	0	0	0	0	0	0	0
Total	421	170	40.38	992	2.36	5	429 (43.25)	474 (47.78)	44 (4.44)	29 (2.92)	16 (1.61)

^a^Flea prevalence = (No. of small mammals with flea infection/No. of small mammals) × 100%.

^b^Flea index = No. of fleas/No. of small mammals.

Among the 9 small mammal species, the flea prevalence of *R. tanezumi* was highest (53.60%, 149/278). The infestation of *R. tanezumi* by *X. cheopis* was particularly high (35.61%, 99/278). *Crocidura attenuata* and *Anourosorex squamipes* were not infested with fleas (Table 3).

**Table 3:** Flea infection of different small mammal species in households of the western Yunnan, China.

Small mammal species	No. of small mammals	No. of small mammals with flea infection	Flea prevalence (%)^a^	No. of fleas	Flea index^b^	No. of small mammals with *X. cheopis*	*X. cheopis* prevalence (%)
*R. tanezumi*	278	149	53.60	737	2.65	99	35.61
*M. musculus*	76	1	1.32	1	0.01	1	1.32
*R. norvegicus*	30	12	40.00	120	4.00	2	6.67
*R. nitidus*	23	2	8.70	4	0.17	1	4.35
*S. murinus*	7	3	42.86	31	4.43	2	28.57
*A. chevrieri*	2	2	100	58	29.00	0	0
*C. attenuata*	2	0	0	0	0	0	0
*A. squamipes*	2	0	0	0	0	0	0
*M.caroli*	1	1	100	41	41	1	1
Total	421	170	40.38	992	2.36	106	25.18

^a^Flea prevalence = (No. of small mammals with flea infection/No. of small mammals) × 100%.

^b^Flea index = No. of fleas/No. of small mammals.

### The results of the prototype multiple HNB regression model

Table 4 shows the prototype multiple HNB regression model for the abundance of fleas. Of the six potential factors, compared to *R. tanezumi,* the probability of flea infestation of *Mus musculus* and other host species decreased, while the number of flea infestations of other host species increased. The number of fleas in adult hosts decreased; the number of fleas in small mammals weighing more than 59 g increased (Table 4).

**Table 4:** The results of the prototype multiple HNB regression model for the abundance of parasitic fleas.

Variables (Factors)	No. of small mammals	Logistic part		Count part	
		OR (95%CI)	*P* value	AR (95%CI)	*P* value
**Host species**					
*R. tanezumi*	270	Ref ^a^		Ref	
*M. musculus*	76	0.01 (0, 0.08)	0	0 (0− + ∞)	0.959
Others	61	0.41 (0.22, 0.76)	0.004	4.40 (1.70, 11.37)	0.002
**Age**					
Immaturity	114	Ref		Ref	
Adult	293	1.71 (0.67, 4.37)	0.265	0.33 (0.10, 1.10)	0.071
**Gender**					
Female	255	Ref		Ref	
Male	152	0.81 (0.51, 1.29)	0.377	1.47 (0.83, 2.61)	0.183
**Body weight (g)**					
≤ 59	202	Ref		Ref	
> 59	205	1.28 (0.54, 3.04)	0.570	5.03 (1.60, 15.82)	0.006
**Body length (cm)**					
≤ 21	205	Ref		Ref	
> 21	202	0.89 (0.52, 1.52)	0.673	1.33 (0.73, 2.44)	0.351
**Tail length (cm)**					
≤ 13	201	Ref		Ref	
> 13	206	0.82 (0.39, 1.72)	0.603	0.55 (0.25, 1.22)	0.140

^a^Ref: reference group.

### The results of the final multiple HNB regression model

The final model showed that four factors—species, age, sex and body weight of small mammals—are closely associated with the abundance of flea (Table 5). Compared to *R. tanezumi*, the probability of flea infestation with *M. musculus* as well as other host species decreased by 58% and 99% respectively, while the number of flea infestations of the other host species increased by 4.71 folds. The probability of flea prevalence in adult hosts increased by 74%, while the number of fleas decreased by 76%. The number of flea infestations in small male mammals increased by 62%. The number of fleas in small mammals weighing more than 59 g increased by about 4 times (Table 5).

**Table 5:** The results of the final HNB model for the abundance of parasitic fleas.

Variables (Factors)	Logistic part		Count part	
	OR (95%CI)	*P* value	AR (95%CI)	*P* value
**Host species**				
*R. tanezumi*	Ref ^a^		Ref	
*M. musculus*	0.01 (0, 0.07)	0.000	0.00 (0− + ∞)	0.956
Others	0.42 (0.23, 0.76)	0.004	5.71 (2.21, 14.77)	0.000
**Age**				
Immaturity	Ref		Ref	
Adult	1.74 (1.07, 2.81)	0.024	0.24 (0.07, 0.80)	0.020
**Gender**				
Female			Ref	
Male			1.62 (0.92, 2.84)	0.093
**Body weight (g)**				
≤ 59			Ref	
> 59			5.03 (1.62, 15.64)	0.005

^a^ Ref: reference group.

## Discussion

In this study, 421 small mammals were captured and *R. tanezumi* was the predominant species. A total of 992 fleas were collected from small mammals. The number of *L. segnis* and *L. cheopis* was accounted for 91.03%. A close relationship between fleas’ abundance and small mammal traits (including species, age, body weight, sex) was evident in households in the western Yunnan province.

The results of our investigation showed that *R. tanezumi* is the predominant species in households in the western Yunnan province. *X. cheopis* and *L. segnis* are dominant fleas, and *R. tanezumi* is their main host. These results are consistent with the previous study that *R. tanezumi* is the dominant rodent species in commensal plague foci, and *X. cheopis* and *L. segnis* are dominant parasitic fleas^23^. In the commensal plague foci of the western Yunnan province, *R. tanezumi* is the main host species, while *M. musculus* is the auxiliary host species. Yin et al.^24^ have suggested that the abundance of fleas is related to host species. Our data indicated that *R. tanezumi* was more likely to be infested with fleas than *M. musculus* and other species. The main host may or may not be the species in which the parasite evolved for the first time, but it’s currently home to most individuals in the parasite population^22^. It is therefore believed that *R. tanezumi* is the best species in which the majority of parasites live and the probability of flea infestation on *M. musculus* as well as other species has decreased compared to *R. tanezumi*. The reason for the increase in the number of fleas in other species could be due to the fact that the number of flea infestations on other species was sufficient, although the number of other host species is lower in other species in our study, like *Apodemus chevrieri*. In addition, the fundamental reasons for the diversity in parasitic abundance between the main host and any auxiliary host are often related to the different successes of exploitation and reproduction of a parasite^25^. Previous studies have shown that the phylogenetic correlation between the main host and the auxiliary host can determine parasite abundance on its auxiliary hosts, as it should reflect the similarities between host species in terms of physiological, ecological characteristics and immunological^26^. On the other hand, it was found that for flea infestation in small mammals, the abundance of a flea on its auxiliary hosts decreased with increasing phylogenetic distance of these hosts relative to the main host^26^. The mechanisms underlying this model are not yet clear but are supposed to be consistent with the differential performance of a flea on auxiliary hosts, which in turn is correlated with a phylogenetic distance from the auxiliary host to the main host^27^.

Recent studies have shown that parasites appear to make favorable choices and decisions for hosts in which their reproductive benefits are maximized^28^. In the meantime, the choice of the host is important, which may contribute to the variation in the abundance of the fleas collected when the potential risk of flea-mediated diseases are assessed. In addition, the variation in the level of infection in host individuals is well known, indicating that some individuals may represent better patches for parasites than other individuals^21^. Namely, reservoirs providing better patches for parasites are usually defined as primary hosts.

Differences in flea infestation due to the age of the host may be influenced by differences in parasite aggregation. We examined the effect of age of small mammals on parasitic fleas and found that the probability of flea prevalence in adult hosts increased but the number of fleas in adult hosts decreased. This phenomenon has generally been linked to host immune responses and parasite-host associations. Firstly, it is known that immune defenses often deteriorate with increasing age^29^, so that in smaller mammals the decline of antiparasitic defense has been reported with increasing age^30^. These hosts have different defenses that can affect the number of blood a flea can acquire^31^. Fleas were found to take more blood from juveniles and older animals than from sub-adult and adult animals^32^. The degree of deterioration of immune function with age may, however, be affected by environmental conditions (e.g. environmental stress)^33^ and may also differ between males and females (due to faster aging of men)^34^. Secondly, differential parasite abundance in hosts belonging to different age cohorts has been reported for various host and parasite taxa^35–37^. However, the influence of host age on the distribution pattern of parasite abundance differs among different host-parasite associations. In some host-parasite associations, the abundance of parasites increases with the age of the host^35^, while in other associations it increases or decreases in the youngest and oldest hosts compared to hosts of median age (called "adult hosts")^36^. In fact, acquired resistance against parasites may be lower in young or old hosts than in median-age hosts. Young hosts may simply not have the time to develop parasite resistance, while older hosts may lose their ability to resist parasites due to immunosenescence^29,38,39^. As a result, hosts in the youngest and the oldest cohorts would have better habitat for parasites, so the relationship between parasite abundance and host age would be convex. If the negative effect of heavy parasite loads causes mortality mainly in young instead of old hosts, the abundance of parasites will increase with the age of the host. In all cases, fleas performed better in younger and older hosts than in middle-aged hosts.

In addition, the effect of the age of the host was strongly influenced by the effect of the sex of the host. In particular, from the point of view of resource acquisition (i.e. The size of the blood meal), there has been an improvement in the quality of cohorts of young and old hosts in females, but not in males, while, in the context of resource processing (digestion of blood), some trends in age-dependent host quality has been observed in male hosts but not in female hosts^31^. In other words, the results of the Liberman et al.^32^ suggest that the age of the host does not predict unequivocally whether it is more or less beneficial for a flea.

Similarly, the sex of the host was also a determining factor influencing the abundance of fleas. Previous studies have shown the effect of host sex in flea reproduction^40,41^. In this study, we studied the consequences of host sex on flea abundance. Finally, we observed an increase in the number of flea infestations in small male mammals compared to females. The effect of host sex on parasite performance is essential to understanding the mechanisms of male-biased parasitism.

Under normal conditions, two non-mutually exclusive mechanisms are invoked to explain the variation in the number of fleas observed. Male hosts are characterized by higher mobility and poorer immunological defense than female hosts. In general, fleas feed faster, take relatively more blood and digest more quickly when feeding on male rather than female, although the pattern of blood digestion related to the sex of the host depends on external conditions (relative humidity). In addition, fleas produced more eggs exploiting male hosts than female hosts^40^. This would lead to a better ability of parasites exploiting male hosts rather than female hosts because of the immunosuppressive effects of androgens, which would lead to fleas favoring male^41^. It is suggested that, in many cases, male hosts represent better patches for parasites than females. Thus, it can be seen that more flea prefers to stay in male hosts than in females. Consequently, a greater infestation of a male host than a female host in terms of abundance, prevalence and species richness of parasites, this has been reported for a wide variety of parasite and host taxa^42^, although higher levels of parasite infection have been reported in some mammalian female^43^.

The body weight of the hosts could be considered as an additional indicator of the number of fleas assembled. The results of this study showed that the number of fleas increased in small mammals weighing more than 59 g. Hawlena^44^ noted that the number of fleas, mainly *X. cheopis* and *Xenopsylla Astia* (Siphonaptera: Pulicidae), caught in the wild (Rodentia: Muridae) increased with the weight of the host (and thus its age) at an optimum before decreasing. In practice, the body weight of the host increases with age. As mentioned above, the probability of flea infestation on the host increases with the age of the host. Therefore, the number of fleas would increase with the body weight of the host.

Associations between the aggregation of fleas and the abundance of their hosts vary according to different factors. This study confirmed that the variation in the number of fleas was due to different factors in the host.

It is concluded that the host is an important factor to consider when comparing fleas variation in natural rodent populations. It is necessary that the factors mentioned above be taken into account to control the abundance of fleas. Fitness related measures should be directly involved, such as reducing the number of small mammals and fleas, preventing and controlling flea-borne diseases.

## Conclusions

The results of the study show the distribution of small mammals and parasitic fleas in households of the western Yunnan province, China. In addition, a new discovery revealed by the hurdle binomial regression model is that there is a close relationship between the abundance of fleas and the characteristics of small mammals (e.g. species, age, sex, and body weight). Our study provides direct evidence to explore the relationship between the fleas and host by the characteristic of instinct and to reveal host structure playing an important role in the allocation of flea abundance in the ecosystem of the plague. The knowledge of flea factors will help control and predict the number of fleas in commensal rodent plague foci and further control the onset of the plague. Although it is relatively limited that the current model predicts the factors affecting the abundance of fleas. In addition to the characteristics of the host, the number of fleas may be affected by other potential factors (e.g. environment, climate, economy, human disturbance). Thus, the above factors should be measured and included in the regression model, it would be useful to establish benefit model to predict the abundance of parasitic fleas.

## Methods

### Description of the study area

The western Yunnan province is located in the central region of the Hengduan mountains of the terrestrial part of the Tibetan plateau, which belongs to the low latitude mountain valley. It is influenced by the enormous difference in latitude and altitude gradient, which is characterized by complex terrain and varied climate^45^. Therefore, it is one of the regions that possess the most abundant animal and plant species in China. There are many minority nationalities of human populations, including Yi, Dai, Naxi, Hui, Bai et al. inhabiting this area. The main economic source is planting and economic development is relatively poor. These conditions contribute to the breeding of small mammals and the spread of the plague.

**Figure 1:**
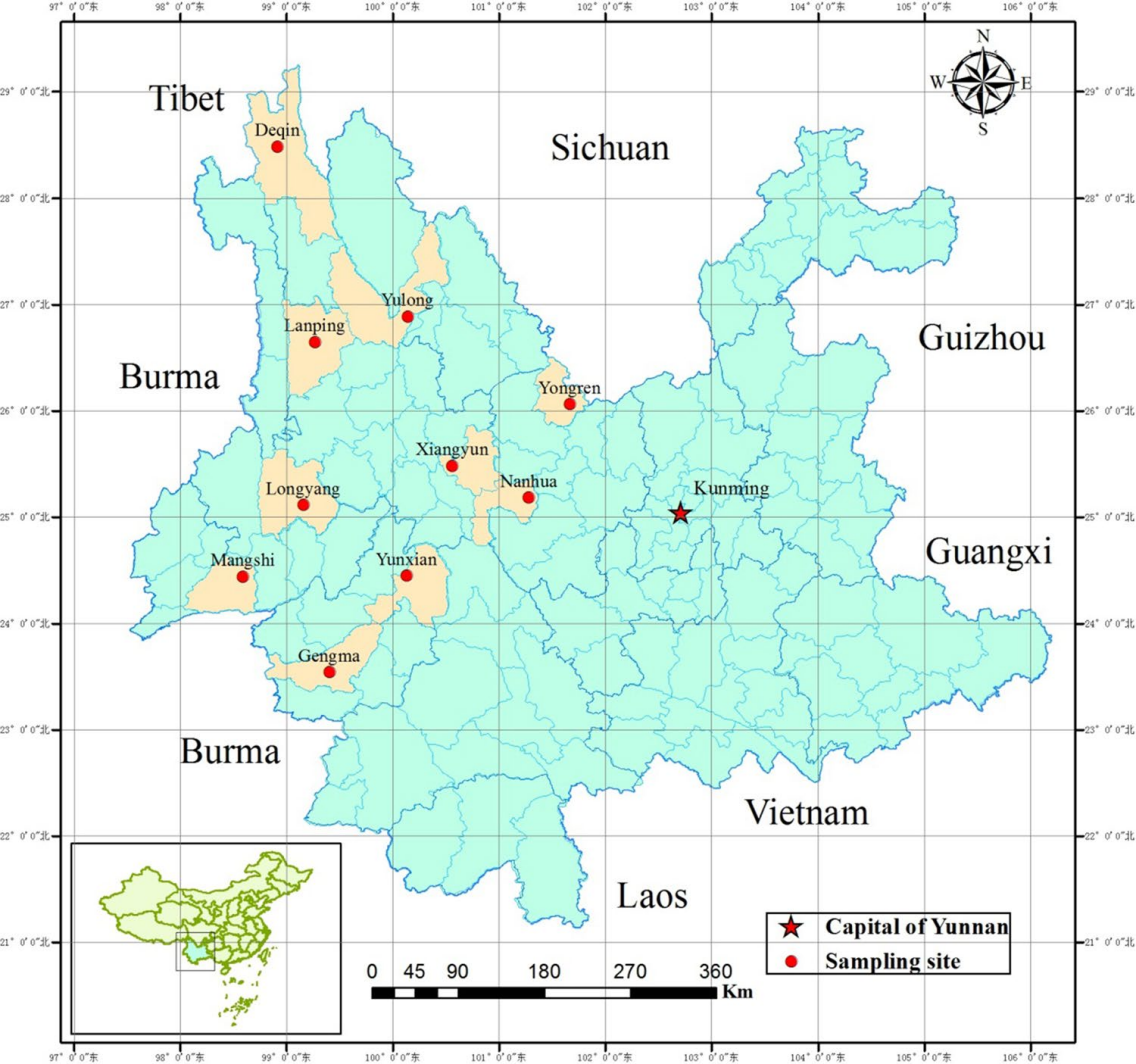
Location of 10 sampling sites in the western Yunnan Province, China. Ten red dots represent 10 sampling counties and yellow shadow is the acreage of each county. Red five-pointed star is the location of Kunming city (capital of Yunnan Province).

The study was carried out in 800 households (Twenty households per village were selected by simple random sampling method) in 40 villages from 10 counties, in the western part of Yunnan province (Fig. 1) (The Fig. 1 was made using QGIS 2.8.1, https://www.qgis.org/en/site/). In the study area, the 10 counties have a total area of ​​approximately 41,130 square kilometers and a human population of approximately 3.4 million.

### Study subjects

The study received ethical approval from the National Natural Science Foundation of China (No. 81860565) and Yunnan Institute of Endemic Disease Control and Prevention (No.YIEDC2011-01). All experiments were performed in accordance with relevant guidelines and regulations.

### Small mammals capture and identification

Five traps were placed in each household for three continuous nights to capture small mammals. The bait was fried ham sausage. Each trap was checked in the morning of the next day. If a small mammal was captured, a new trap with fresh bait was replaced in the same place. The species of small mammals, sex and age were identified according to their morphological characteristics. The age of small mammals was identified by the development of the reproductive organs, teeth, and the body weight^46^. Their weight, body length and tail length were measured.

### Fleas collections and identification

The fleas were harvested by combing from the tail end forward using a toothbrush in a white enamel tray after anesthetizing small mammals with ether. The fleas were stored in labeled vials containing 75% ethanol. Fleas from each small mammal were kept in separate vials and kept at room temperature. After the fleas were taken from vials and rinsed with 0.9% normal saline, the species was identified under a light microscope based on the external morphology of the fleas^47^.

### Data analysis

The distribution of small mammals and fleas was summarized using descriptive statistics. Then the flea prevalence and the abundance of fleas of each small mammal were generated^48^. Species, sex, age, weight, body length and tail length of each small mammal were considered potential factors for the abundance of fleas. For continuous variables, the median was used to classify these variables into binary variables. The relationship between potential factors and a result was explored using the hurdle negative binomial (HNB) regression model using R software 3.02^49–51^. This regression model was applied to take into account of the current dataset that shows a count of excess zeros and an overdispersion distribution. It is a two-component model: one is the logistic model adjusting counts against zero, the other is the hurdle binomial model adjusting positive counts. The factors with *P* < 0.20 in univariate analysis were regarded as the potential factors related to fleas and entered the prototype multiple HNB regression model. The final model to predict the factors associated with the flea abundance was refined using a backward method (α = 0.10 as a criterion of statistical significance). The proportion of prevalence odds ratio (OR) and abundance ratio (AR) for a parasitic infestation of fleas were calculated based on pieces of literature^24,52^.

### Ethical statement

The study has been approved by the National Natural Science Foundation of China (No. 81860565) and Yunnan Institute of Endemic Disease Control and Prevention (No. YIEDC2011-01).
